# Effects of blood flow restriction training on anthropometric and blood lipids in overweight/obese adults: Meta-analysis

**DOI:** 10.3389/fphys.2022.1039591

**Published:** 2022-11-29

**Authors:** Lei Sun

**Affiliations:** School of Sport Sciences, Nanjing Normal University, Nanjing, China

**Keywords:** anthropometric index, blood lipid, meta-analysis, overweight, obesity

## Abstract

**Abstract:** Purpose: To systematically evaluate the effects of blood flow restriction training (BFRT) on anthropometric indicators and blood lipids in overweight/obese adults.

**Methods:** A literature search was conducted on PubMed, Web of Science, Embase, Scopus, SPORTDiscus and Cochrane Library databases to determine the final literature based on inclusion and exclusion criteria. Review Manager 5.4.1 was used to evaluate the quality of the literature based on the Cochrane bias risk assessment tool, and Stata 17.0 software was used for Meta-analysis.

**Results:** A total of 3,985 articles were screened, and five of the studies were included in the Meta-analysis, with a total 66 participants. In each study, subjects were measured before and after BFRT. Meta-results showed that BFRT significantly reduced BMI, lowered body weight, body fat % and waist circumference, significantly reduced total cholesterol (TC) and low-density lipoprotein cholesterol (LDL-C) level, lowered triglycerides, and increased high-density lipoprotein cholesterol (HDL-C) level in overweight/obese adults.

**Conclusion:** BFRT can be used as a safe and effective exercise prescription for personalized weight/fat loss. BFRT significantly reduces BMI by reducing body weight, body fat %, and waist circumference and has the effect of improving body composition. It also significantly reduced TC and LDL-C and tends to decrease TG and increase HDL-C in overweight/obese adults, potentially reducing the incidence of cardiovascular disease.

## 1 Introduction

In recent years, the prevalence of overweight/obesity has been on the rise in both developed and developing countries and has become a global public health problem (Lang and Froelicher, 2006). In China, the United States, Canada and the United Kingdom, the incidence of overweight/obesity exceeds 20% of the total population (Flegal et al., 2013). The World Health Organization considers overweight/obesity to be one of the world’s most serious public health problems. The European Commission regards overweight/obesity as a chronic disease, and its occurrence is often accompanied by an increase in the incidence of chronic diseases such as hyperlipidemia, type 2 diabetes, atherosclerosis, liver disease, cancer and neurodegenerative diseases. The impact is wide ranging affecting 6.7 billion people worldwide and is growing at a threefold rate. This leads to increased mortality and people becoming pessimistic about the potential success of treatments (Flegal et al., 2013; Saltiel and Olefsky, 2017; Burki, 2021; Teufel et al., 2021).

In the management of adult overweight/obesity, moderate-intensity aerobic exercise can reduce body weight, total fat, visceral fat, intrahepatic fat, and control blood pressure, whereas moderate-intensity resistance exercise can maintain lean body mass during weight loss (Oppert et al., 2021). However, for people with high body weight/obesity, the elderly, recovering athletes, or the general population without training experience (who cannot withstand the high mechanical pressure on the joints during high resistance training), performing high intensity strength or aerobic training may lead to the occurrence of sports injury. These risks are highlighted in a number of publications. For instance, high body mass index and percent body fat can significantly affect the markers of muscle injury after high intensity exercise (Kim and Yoon, 2021), and high intensity of swimming, bicycling, and running can cause muscular damage in athletes (Huang et al., 2019). It has also been shown that moderate and high-intensity walking for training may induce orthopedic, leg, foot and groin injury in the elderly (Carroll et al., 1992) and among untrained women and men. A single bout of maximal eccentric exercise of the elbow flexors may result in greater vulnerability to injury (Hubal et al., 2008). Significantly, blood flow restriction training can promote recovery of the musculoskeletal system (Hughes et al., 2017) and rehabilitation of knee injuries (Li et al., 2021). Therefore, low-intensity training combined with KAATSU training or vascular occlusion training was developed. Blood flow restriction training (BFRT) involves applying external pressure to the limb during exercise. This is done using special devices such as inflatable cuffs or elastic bandages, or using compression wraps such as a blood pressure cuff or other specially designed restraint bands. BFRT is a method to achieve the effect of intensive training by partially blocking arterial blood flow and occlusion of venous blood flow of the pressured limbs (Wortman et al., 2021). BFRT combined with low intensity resistance or endurance training can lead to an increase in muscle strength and mass. As well, blood flow restriction combined with endurance training has also been shown to improve cardiopulmonary health (Conceição and Ugrinowitsch, 2019). Furthermore, BFRT can effectively improve muscle mass and strength in different populations (Davids et al., 2021; Korkmaz et al., 2022), improve muscle fitness (Hill et al., 2021), increase aerobic capacity (Billaut et al., 2022), promote knee rehabilitation (Centner et al., 2019), stimulate acute bone formation markers and hormonal responses (Bemben et al., 2022), and improve athletic performance thereby maintaining overall good health (Wortman et al., 2021). However, in the field of BFRT intervention for overweight/obesity, studies on anthropometric indicators and blood lipids have shown different results (Da Silva et al., 2020; Kim and LeeDongminWon, 2021; Mohammadiyan et al., 2021; Yong et al., 2021). Studies have shown that BFRT significantly improves anthropometric indicators (body weight (BW), body fat percentage (BF%), BMI and waist circumference (WC)) and blood lipids (triglyceride (TG), total cholesterol (TC), low-density lipoprotein cholesterol (LDL-C), and high-density lipoprotein cholesterol (HDL-C)) (Yong et al., 2021; Razi et al., 2022). However, some studies found that anthropometric indicators (BW, BF%, BMI and WC) and blood lipids (TG, TC, LDL-C, and HDL-C) did not show significant changes (Da Silva et al., 2020; Mohammadiyan et al., 2021). Although there are relevant randomized controlled trial studies, the results are inconsistent due to limited sample size, gender difference, age ranges and other factors, so it is difficult to judge the effect of BFRT. Therefore, it is necessary to conduct an integrated analysis of this type of study.

This study conducted a Meta-analysis by referring to the effect of BFRT on overweight/obese people and evaluated the heterogeneity among studies. It focused on anthropometric indicators (BW, BF%, BMI, and WC) and blood lipids (TG, TC, LDL-C, and HDL-C) aiming to provide practical reference for BFRT to control anthropometric indicators and blood lipids in overweight/obese adults.

## 2 Methods

### 2.1 Retrieval strategy and data sources

The meta-analysis priority report item was based on PRISMA guidelines (Supplementary Table S1) (Moher et al., 2009). Two researchers used subject words combined with free words to conduct a literature retrieval using an independent double-blind method. In this review, Population: The studies that were conducted in overweight/obese population; Intervention: BFRT that led to improve the dental health status of the target groups; The comparison group: Participants that did not receive BFRT interventions; Outcomes: Interventions that led to the improvement of outcomes in anthropometric and blood lipids. Studies: Randomized trial studies, pretests and post-tests, experimental studies. A literature search was performed on PubMed, Web of Science, Embase, Scopus, SPORTDiscus and Cochrane Library databases by title, abstract and keywords (Supplementary Table S2). Boolean operators “AND” and “OR” were used to conduct database searches and included the following key English terms (“blood Flow Restriction Therapy” OR “BFR Therapy” OR “BFR Therapies” OR “Therapy, BFR” OR “blood Flow Restriction Training” OR “blood Flow Restriction Exercise” OR “blood flow restriction” OR “blood flow restricted” OR “kaatsu” OR “tourniquets” OR “ischemia” OR “vascular occlusion” OR “occlusion training”) AND (“overweight” OR “obesity” OR " adiposity” OR “appetite depressants” OR “body weight” OR “diet, reducing” OR “skinfold Thickness” OR “lipectomy” OR “anti-obesity Agents” OR “bariatrics”) AND (“randomized controlled trial” OR “randomized” OR “placebo” OR “RCT”). The retrieved literatures were screened by duplication, title and abstract, and read full text to determine the final inclusion. The last retrieval time was June 2022.

### 2.2 Inclusion and exclusion criteria

This study focused on the effect of BFRT on anthropometric indicators and blood lipids in overweight/obese adults. Inclusion criteria was according to PICOS principles. The inclusion criteria were: 1) Subjects: regardless of nationality, region and gender; 2) Intervention: BFRT; 3) Outcome indicators: anthropometric indicators (BW, BF%, BMI, and WC) and blood lipids (TG, TC, HDL-C, and LDL-C); 4) Literature type: Randomized controlled trial (RCT) include parallel and crossover trials, no matter blind, assignment hidden, or lost to follow-up.

The literature exclusion process was conducted by two researchers independently. The retrieved literature hid authors, institutions, and published journals titles. For inconsistent results, further discussion or third-party adjudication was adopted. The exclusion criteria were: 1) If the article type of the article is meta or a review, it will be excluded; 2) If the research object of the article is animal experiment, it will be excluded; 3) Conference abstracts, case reports, and investigations will be excluded; 4) If the research object of the article is the disease model, it will be excluded; 5) Articles written in Chinese will be excluded; 6) The research results of the article will be excluded if there is no common output result; 8) People who are not overweight/obese and have physical diseases will be excluded. Exclusion reasons of full tests are in Supplementary Table S3.

### 2.3 Intervention characteristics

Included studies should involve intervention protocol who performing BFRT combined different forms of exercise. The BFRT protocol was used in all the articles supplemented with a reproducible and reliable method of restricting blood-flow to the working muscles with a blood pressure cuff, mainly in the proximal thighs or arms of the upper limbs (sphygmomanometer tape, surgical tourniquet, KAATSU equipment, etc.). Before the intervention, the participants took a familiarization session according to their experimental group to get acquainted with the training protocol and testing procedures and the pressure of the cuff was increased until the participants adapted to the restriction stimulus during the early phase of training and the relative pressure was maintained used during the training protocol.

### 2.4 Literature quality evaluation

Cochrane risk bias assessment tools were used by two researchers respectively to evaluate the risk of the included literature, which includes selection bias, performance bias, detection bias, attrition bias, reporting bias, and other biases. The judgment criteria were high risk bias, low risk bias and uncertainty bias. If there are differences of opinion, three people need to discuss to reach an agreement.

### 2.5 Data extraction

Get Data2.25 software was used to extract the Mean and standard deviation (SD) data of the histogram of outcome indicators in the study of Kim et al. (2021). First, pictures are opened in the article by software. Next, the coordinate system is set, and the minimum and maximum values of *X* and *Y* axes are set. After that, the point capture mode picks up the data points, and finally the data is exported. Excel 2019 was used to summarize the Mean and SD, which measured before and after the outcome indicators of five studies. Two researchers used an independent double-blind method to extract relevant indicators of the included literature including: researchers, publication year, age, gender, interventions, intensity, frequency, duration, outcome indicators. Data information was obtained by translating literatures Korean Kim et al. (2021) and Arabic Mohammadiyan et al. (2021).

### 2.6 Data analysis

Literature quality was evaluated by Revman 5.4.1, and Stata 17.0 was used for heterogeneity identification and effect size combination. The included data were continuous outcome variables with the same measurement units, and their effect sizes were weighted mean difference (WMD) and 95% confidence interval (CI). According to the Cochrane classification, I^2^ was used to identify the heterogeneity between studies. Mild heterogeneity (0%–40%), moderate heterogeneity (40%–60%), large heterogeneity (50%–90%), and large heterogeneity (75%–100%) were acceptable. Fixed effect model was used for low heterogeneity or *p* > 0.01, and random effect model was used for large heterogeneity or *p* ≤ 0.01. Statistical significance was set at *p* < 0.05.

## 3 Results

### 3.1 Literature retrieval and screening

**FIGURE 1:**
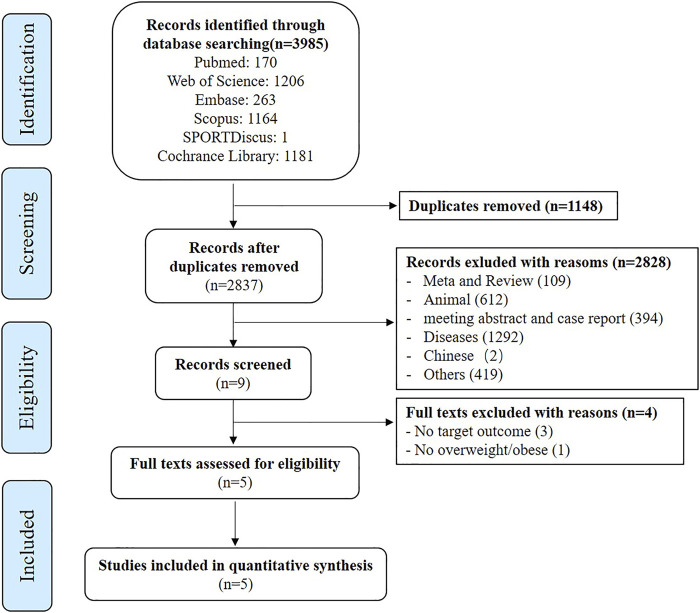
Flow diagram demonstrating the step by step process of article elimination to find the final articles to be included.

All databases were searched preliminarily, and a total of 3,985 articles were included. According to the inclusion and exclusion criteria, a total of 3,980 articles were excluded through screening and reading of title, abstract and full texts, and finally five articles were included (Figure 1).

### 3.2 Characteristics of the included literature

The five included studies included BFRT, a total of 66 participants, mean age range was distributed by age 25-year-old. The results of the included literatures were tested before and after BFRT. The design of each experimental scheme was flexible, and training duration was 5–18 weeks, of which 8 and 12 weeks were the majority. All recruited participants were untrained and healthy. (Table 1).

**TABLE 1:** Basic information of the included literatures.

Study	Year	Age	Gender	Intervention	Intensity	Frequency	Duration	Outcome
			(Male/female)					
da et al. Da Silva et al. (2020)	2020	25.52 ± 2.19	15/0	LLRT + BFRT	30% 1RM	3 times/week	8 weeks	BW, BF%, BMI, WC, TG, TC, LDL-C, HDL-C
Kim et al. Kim and LeeDongminWon. (2021)	2021	22.3 ± 1.0	0/9	LIRT + BFRT	40% 1RM	2 times/week	5 weeks	BW, BF%, BMI, TG, TC, LDL-C, HDL-C
Chen et al. Yong et al. (2021)	2021	20.3 ± 1.07	18/0	LIC + BFRT	40% VO2max	2 times/week	12 weeks	BW, BF%, BMI, WC, TG, TC, LDL-C, HDL-C
Razi et al. (Razi et al. (2022)	2022	44.77 ± 5.09	9/0act	Walking + BFRT	5–10min, 3 km/h	3 times/week	8 weeks	TG, LDL-C, HDL-C
Mohammadiyan et al. (Mohammadiyan et al. (2021)	2021	27.41 ± 5.17	0/15	LIRT + BFRT	20%–30% 1RM	3 times/week	6 weeks	BW, BF%, BMI, WC

Note:BW = body weight; BF% = body fat percentage; WC = waist circumference; TG = triglyceride; TC = total cholesterol; LDL-C = low-density lipoprotein cholesterol; HDL-C = High-density lipoprotein cholesterol; LIRT = low intensity resistant training; LIC = low intensity cycling.

### 3.3 Literature quality evaluation

**FIGURE 2:**
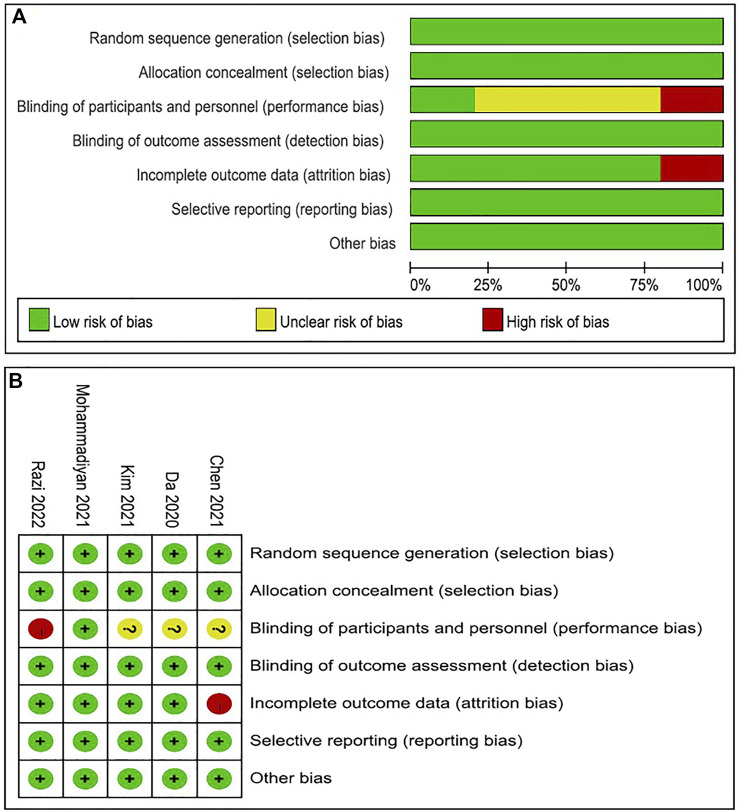
Risk-of-bias assessment. **(A)** Risk of bias summary; **(B)** Overall risk of bias.

The quality of the random sequence generation and selective outcomes report of the five literatures were all low-risk (Figure 2). High risk in blind execution of research, completeness of outcome measures, including high risk of loss of follow-up or withdrawal. When the informed consent was signed, some studies directly informed the subjects of the study group and purpose, which resulted in high risk. It is a basic requirement of each RCT experiment to use a blind method when processing the result index. In addition, these analyses also included association of the PEDro scale (Table 2). Regarding methodological quality, five studies scored ≥8 on the PEDro scale. The main limitation of these five studies was blinding of subjects, therapists and assessors. Based on the five included studies it was not deemed meaningful to perform a funnel plot, nor perform an Egger’s test.

**TABLE 2:** Quality assessment of included Studies—PEDro scale items.

First authors	Da (2020)	Kim (2021)	Chen (2021)	Razi (2022)	Mohammadiyan (2021)
A	1	1	1	1	1
B	1	1	1	1	1
C	1	1	1	1	1
D	1	1	1	1	1
E	0	0	0	0	1
F	0	0	0	0	0
G	0	0	0	0	0
H	1	1	1	1	1
I	1	1	1	1	1
J	1	1	1	1	1
K	1	1	1	1	1
Total	8	8	8	8	9

Abbreviations: PEDro, scale items (each satisfied item except the first item contributes one point to the total PEDro, score): A, eligibility criteria; B,randomization; C, allocation concealment; D, similar at baseline; E, blinded subjects; F, blinded therapist; G, blinded assessors; H, <15% dropouts; I. ITT, analysis; J, between-group comparison; K, point and variability measures; 1, item positive; 0, item negative or unknown.

### 3.4 Meta-analysis

#### 3.4.1 The effect of BFRT on anthropometric indicators

**FIGURE 3:**
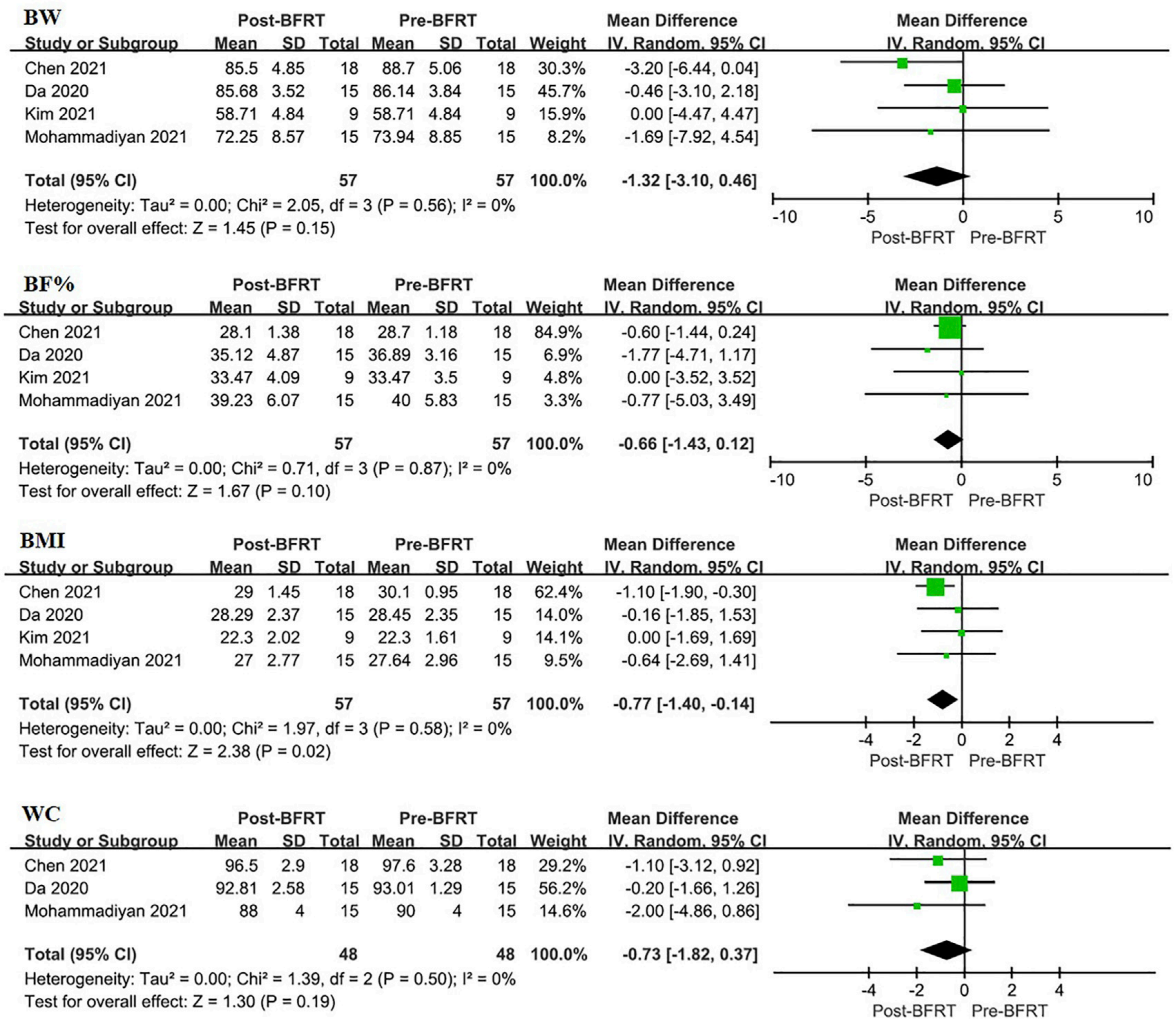
Forest plot displaying the difference in anthropometric indicators between experimental group and control group for each individual case. BW, body weight; BF%, body fat percentage; WC, waist circumference.

A Meta-analysis was conducted on studies involving anthropometric indicators (Figure 3). The results of forest map using fixed effect model, which indicated that BFRT had a better impact on anthropometric indicators of overweight/obese adults than before. BFRT was associated with BW (MD = −1.32, 95%CI: −3.10–0.46, *p* = 0.15), BF% (MD = −0.66, 95%CI: −1.43–0.12, *p* = 0.10) and WC (MD = −0.73, 95%CI: −1.82∼ −0.37, *p* = 0.19) had an improvement effect but not significant, only for BMI (MD = −0.77, 95%CI: −1.40∼ −0.14, *p* = 0.02). Heterogeneity identification showed that BW (I^2^ = 0%, *p* = 0.53), BF% (I^2^ = 0%, *p* = 0.87), BMI (I^2^ = 0%, *p* = 0.58) and WC (I^2^ = 0%, *p* = 0.50) showed good homogeneity among included studies.

#### 3.4.2 The effect of BFRT on blood lipids

**FIGURE 4:**
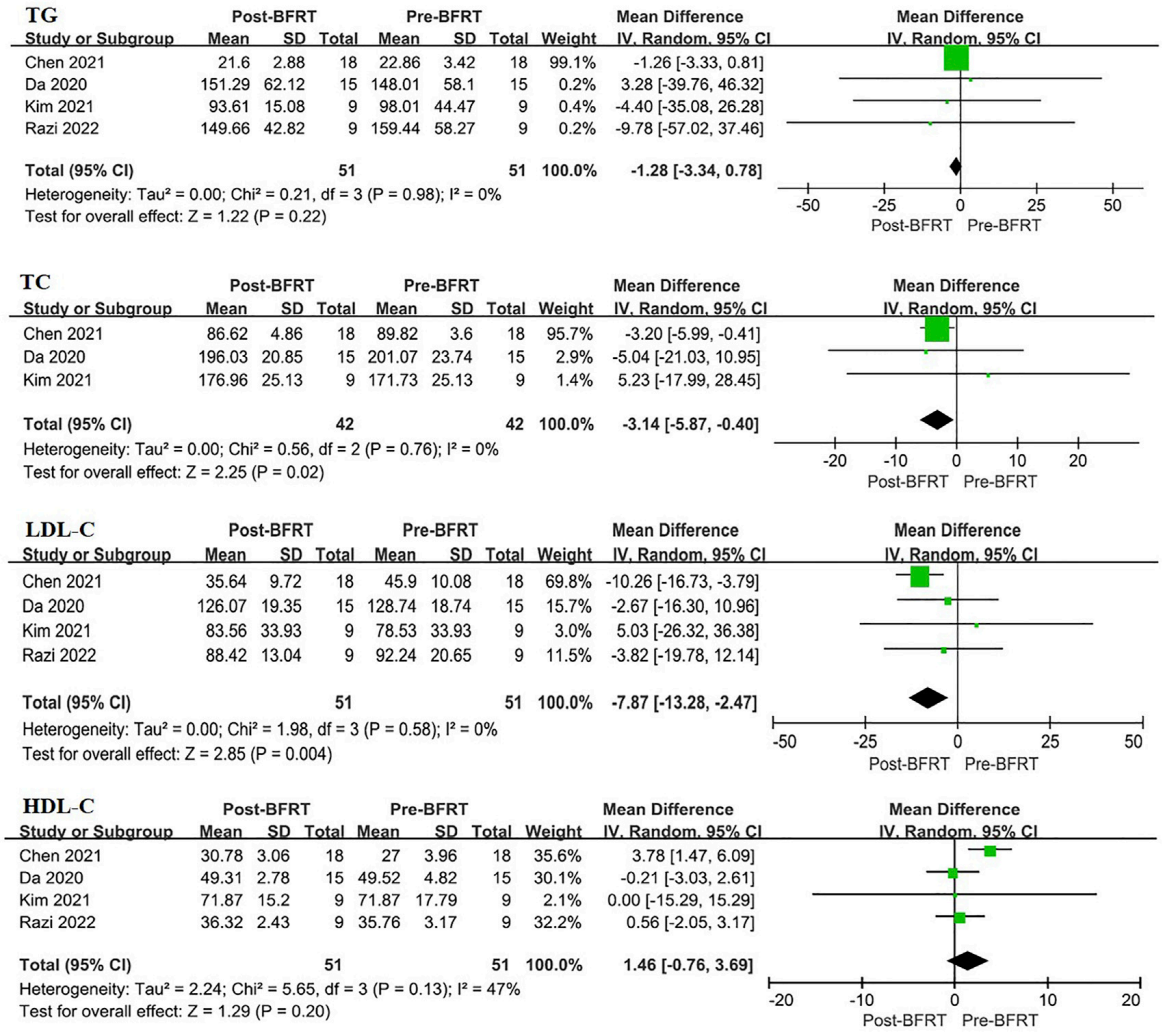
Forest plot displaying the difference in blood lipids between experimental group and control group for each individual case. TG, triglyceride; TC, total cholesterol; LDL-C, low-density lipoprotein cholesterol; HDL-C, high-density lipoprotein cholesterol.

Meta-analysis was conducted on the studies involving blood lipids (Figure 4). Forest map results showed that BFRT had a better effect on blood lipids in overweight/obese adults than before. BFRT had a better effect on TC (MD = −3.14, 95%CI: −5.87–0.40, *p* = 0.02) and LDL-C (MD = −7.78, 95%CI: −13.28∼ −2.47, *p* = 0.004), but not HDL-C (MD = 1.46, 95%CI: −0.76–3.69, *p* = 0.20) and TG (MD = −1.28, 95%CI: −3.34–0.78, *p* = 0.22). Through heterogeneity identification, TG (I^2^ = 0%, *p* = 0.98), TC (I^2^ = 0%, *p* = 0.76), LDL-C (I^2^ = 0%, *p* = 0.58) and HDL-C (I^2^ = 47%, *p* = 0.13) showed good homogeneity among included studies.

## 4 Discussion

To our knowledge, this is the first systematic review investigating the pooled effects of BFRT interventions on anthropometric outcomes and blood lipids and is also the first to compare these effects before and after BFRT in overweight/obese adults. The main findings of this meta-analysis suggest that BFRT is an effective intervention to improve body weight and lower BMI significantly, meanwhile also decreasing TC and TG in overweight/obese adults. In addition, the meta-analysis revealed that BFRT tends to decrease BW, BF%, WC, TG and increase HDL-C level but not to a significantly different level, whereas too few studies are currently available examining the effect of BFRT in overweight/obese adults.

Epidemiological studies commonly use BMI as an indicator of overweight/obesity. The definition of overweight and obesity in adults in the United States and the world, which is based on BMI. The basic assumption of using BMI to define obesity is that, at a given height, the higher the BW, the higher degree of obesity (Flegal et al., 2009). However, BMI is not a perfect measure of body obesity, mainly because it does not directly measure fat mass (Roche et al., 1981). For example, when determining obesity from BMI, it correctly identified about 44% of obese men and 52% of obese women (Roche et al., 1981). The same applies to overweight, which is also determined by a BW/height^2^ (kg/m^2^). However, obesity is due to the physical changes that occurs after becoming overweight, mainly reflected in BMI and body fat changes, many changes in body composition also occur with changes in body fat content, which is particularly important because being overweight is associated with an increase in lean mass (Caballero, 2019). So, a single evaluation index is not enough to determine overweight/obesity. Therefore, a receiver operating characteristic (ROC) score of BF% combined with a BMI of 25 kg/m^2^ for men and 23 kg/m^2^ for women was used as a diagnostic screening threshold for obesity specially (Wellens et al., 1996; Kondo et al., 2009). In addition, it has been found that WC can be used as an indicator of obesity-related health risks, while BMI can still be used as an important predictor of overweight/obesity health risks when WC is normal or high (Janssen et al., 2004). It indicated that BW, BF%, BMI, and WC are used as diagnostic indicators of overweight/obesity, and the control of these indicators comprehensively can be used as effective strategies to prevent and monitor overweight/obesity (Flegal et al., 2009; Petridou et al., 2019; Wang et al., 2021). In addition, it is necessary to define overweight/obesity by BMI combined with other indicators such as WC or BF%, rather than a single indicator. This will also reduce the occurrence of misdiagnosis, so the evaluation criteria of future national physical fitness tests need to be further refined.

In the course of overweight/obesity, the most typical lipid abnormalities include changes in blood routine measurement parameters, namely the increase of TG and TC, especially the increase of LDL-C and the decrease of HDL-C (Mika and Sledzinski, 2017). Studies have shown that reduced lipid levels, including TG, TC, HDL-C, and LDL-C, are associated with weight loss and reduced risk of many noncommunicable diseases, such as cardiovascular disease (Mika and Sledzinski, 2017). First, since the ability of TG production is critical for fat accumulation and their synthesis is believed to occur through a single mechanism, inhibition of triglyceride synthesis can be a potential target for obesity treatment (Chen and Farese, 2000). Previous studies have proved that 88 mg/dl (1.0 mmol/L) plasma TG are associated with an increased risk of cardiovascular disease in approximately 30% of men and 75% of women (Cullen, 2000). To explore the potential mechanism of its influence, which have revealed that apolipoprotein APOA5 stimulates intravascular VLDL-TG hydrolysis by activating lipoprotein enzymes to increase the absorption of free fatty acids generated by TG hydrolysis in muscle and adipose tissue and eliminate TG in blood (Wu et al., 2010). Of course, triglycerides are also used by the body for energy, and are involved in the body’s energy metabolism. Therefore, it is important to maintain TG balance in the blood. In addition, serum TC can cause atherosclerosis, and which is from LDL-C, and cholesterol imbalance is considered to be a feature of fat cell enlargement in obesity, and the synthesis rate of cholesterol in fat is only 4% of liver, so most fat cholesterol comes from circulating lipoprotein (Yu et al., 2010). In other words, cholesterol can be obtained from lipoproteins, which are found in the circulatory system by binding to cholesterol. And these two lipoproteins are LDL and HDL, which are responsible for transporting cholesterol, and LDL binds cholesterol to transport it to extra-hepatic tissues in the form of LDL-C, and HDL binds cholesterol back to the liver in the form of HDL-C (Son et al., 2015). In the circulation, the increase of TG and TC, the decrease of HDL-C and the increase of LDL-C level are the main characterization indicators of cardiovascular disease (Mika and Sledzinski, 2017). In our study, it showed that BFRT significantly reduced serum TC and LDL-C levels, and HDL-C tended to increase, and TC tended to decrease in overweight/obese adults (Figure 4), which may reduce the incidence of cardiovascular diseases.

Limitations and prospects of this study: 1) There are only a small number of literatures meeting the inclusion requirements, most lacking large-scale, continuous and in-depth clinical studies. It is expected that more studies will further expand the meta-analysis results of this part in the future, and provide more theoretical support for BFRT intervention in cases of overweight/obesity; 2) The included literature failed to elaborate on specific flow limiting pressure, but only fluctuates in a specific range, therefore, the blood flow limiting pressure should be fixed to facilitate the determination of the optimal blood flow limiting pressure; 3) The subjects were mostly men, and women were excluded from the study due to physiological factors. Therefore, women should be included in the study to distinguish gender differences in BFRT intervention; 4) The number of subjects was small; therefore, the sample size should be increased to improve the credibility of the results; 5) Not distinguishing BFRT binds to different forms of exercise due to small groups, therefore groups of different forms of exercise should be performed in the future.

## 5 Conclusion

BFRT can be used as a safe and effective exercise prescription for personalized weight/fat loss. BFRT significantly reduced BMI, a tendency to decrease BW, BF%, and WC, and has a certain improvement effect on body composition. Additionally, BFRT significantly decreased TC and LDL-C level, also tends to decrease TG and increase HDL-C levels in overweight/obese men adults, which may have the potential to reduce the incidence of cardiovascular disease.

## 6 Perspectives

As concluded in this meta-analysis BFRT appears to represent a valid alternative in overweight/obesity adults. Our findings support that BFRT intervention is effective exercise prescription to keep anthropometric indicators and blood lipids to a heathy measurement range in overweight/obese adults, which may have the potential to reduce the incidence of cardiovascular disease.

## Data Availability

The original contributions presented in the study are included in the article/Supplementary Material, further inquiries can be directed to the corresponding author.
