# Supplementary material for: Effects of blood flow restriction training on anthropometric and blood lipids in overweight/obese adults: Meta-analysis
Source: Front Physiol. 2022 Nov 29;13:1039591. doi: 10.3389/fphys.2022.1039591 (PMC9745437; doi:10.3389/fphys.2022.1039591)
Supplement: Supplementary file 1 [file Table1.docx]

**Table S1 Search strategies**

|  | Search number | Query |
| --- | --- | --- |
| Pubmed | 4 | ((((((((((((((blood Flow Restriction Therapy[Title/Abstract]) OR (BFR Therapy[Title/Abstract])) OR (BFR Therapies[Title/Abstract])) OR (Therapy, BFR[Title/Abstract])) OR (blood Flow Restriction Training[Title/Abstract])) OR (blood Flow Restriction Exercise[Title/Abstract])) OR (blood flow restriction[Title/Abstract])) OR (blood flow restricted[Title/Abstract])) OR (Kaatsu[Title/Abstract])) OR (Tourniquets[Title/Abstract])) OR (Ischemia[Title/Abstract])) OR (vascular occlusion[Title/Abstract])) OR (occlusion training[Title/Abstract])) AND ((((((((((Overweight[Title/Abstract]) OR (Obesity[Title/Abstract])) OR (Adiposity[Title/Abstract])) OR (appetite depressants[Title/Abstract])) OR (body weight[Title/Abstract])) OR (diet, reducing[Title/Abstract])) OR (skinfold Thickness[Title/Abstract])) OR (Lipectomy[Title/Abstract])) OR (anti-obesity Agents[Title/Abstract])) OR (Bariatrics[Title/Abstract]))) AND ((((randomized controlled trial[Title/Abstract]) OR (Randomized[Title/Abstract])) OR (Placebo[Title/Abstract])) OR (RCT[Title/Abstract])) |
|  | 3 | (((randomized controlled trial[Title/Abstract]) OR (Randomized[Title/Abstract])) OR (Placebo[Title/Abstract])) OR (RCT[Title/Abstract]) |
|  | 2 | (((((((((Overweight[Title/Abstract]) OR (Obesity[Title/Abstract])) OR (Adiposity[Title/Abstract])) OR (appetite depressants[Title/Abstract])) OR (body weight[Title/Abstract])) OR (diet, reducing[Title/Abstract])) OR (skinfold Thickness[Title/Abstract])) OR (Lipectomy[Title/Abstract])) OR (anti-obesity Agents[Title/Abstract])) OR (Bariatrics[Title/Abstract]) |
|  | 1 | ((((((((((((blood Flow Restriction Therapy[Title/Abstract]) OR (BFR Therapy[Title/Abstract])) OR (BFR Therapies[Title/Abstract])) OR (Therapy, BFR[Title/Abstract])) OR (blood Flow Restriction Training[Title/Abstract])) OR (blood Flow Restriction Exercise[Title/Abstract])) OR (blood flow restriction[Title/Abstract])) OR (blood flow restricted[Title/Abstract])) OR (Kaatsu[Title/Abstract])) OR (Tourniquets[Title/Abstract])) OR (Ischemia[Title/Abstract])) OR (vascular occlusion[Title/Abstract])) OR (occlusion training[Title/Abstract]) |
| Web of science | 1 | blood Flow Restriction Therapy (TS) OR BFR Therapy (TS) OR BFR Therapies (TS) OR Therapy, BFR (TS) OR blood Flow Restriction Training (TS) OR blood Flow Restriction Exercise (TS) OR blood flow restriction (TS) OR blood flow restricted (TS) OR Kaatsu (TS) OR Tourniquets (TS) OR Ischemia (TS) OR vascular occlusion (TS) OR occlusion training (TS) |
|  | 2 | Overweight (TS) OR Obesity (TS) OR Adiposity (TS) OR appetite depressants (TS) OR body weight (TS) OR diet, reducing (TS) OR skinfold Thickness (TS) OR Lipectomy (TS) OR anti-obesity Agents (TS) OR Bariatrics (TS) |
|  | 3 | randomized controlled trial (TS) OR Randomized (TS) OR Placebo (TS) OR RCT (TS) |
|  | 4 | #3 AND #2 AND #1 |
| Embase | #31 | ('blood flow restriction training':ab,ti OR 'blood flow restriction therapy':ab,ti OR 'bfr therapy':ab,ti OR 'bfr therapies':ab,ti OR 'therapy, bfr':ab,ti OR 'blood flow restriction exercise':ab,ti OR 'blood flow restriction':ab,ti OR 'blood flow restricted':ab,ti OR 'kaatsu':ab,ti OR 'tourniquets':ab,ti OR 'ischemia':ab,ti OR 'vascular occlusion':ab,ti OR 'occlusion training':ab,ti) AND ('overweight':ab,ti OR 'obesity':ab,ti OR 'adiposity':ab,ti OR 'appetite depressants':ab,ti OR 'body weight':ab,ti OR 'diet, reducing':ab,ti OR 'skinfold thickness':ab,ti OR 'lipectomy':ab,ti OR 'anti-obesity agents':ab,ti OR 'bariatrics':ab,ti) AND ('randomized controlled trial':ab,ti OR 'randomized':ab,ti OR 'placebo':ab,ti OR 'rct':ab,ti) |
|  | #30 | 'randomized controlled trial':ab,ti OR 'randomized':ab,ti OR 'placebo':ab,ti OR 'rct':ab,ti |
|  | #29 | 'rct':ab,ti |
|  | #28 | 'placebo':ab,ti |
|  | #27 | 'randomized':ab,ti |
|  | #26 | 'randomized controlled trial':ab,ti |
|  | #25 | 'overweight':ab,ti OR 'obesity':ab,ti OR 'adiposity':ab,ti OR 'appetite depressants':ab,ti OR 'body weight':ab,ti OR 'diet, reducing':ab,ti OR 'skinfold thickness':ab,ti OR 'lipectomy':ab,ti OR 'anti-obesity agents':ab,ti OR 'bariatrics':ab,ti |
|  | #24 | 'bariatrics':ab,ti |
|  | #23 | 'anti-obesity agents':ab,ti |
|  | #22 | 'lipectomy':ab,ti |
|  | #21 | 'skinfold thickness':ab,ti |
|  | #20 | 'diet, reducing':ab,ti |
|  | #19 | 'body weight':ab,ti |
|  | #18 | 'appetite depressants':ab,ti |
|  | #17 | 'adiposity':ab,ti |
|  | #16 | 'obesity':ab,ti |
|  | #15 | 'overweight':ab,ti |
|  | #14 | 'blood flow restriction training':ab,ti OR 'blood flow restriction therapy':ab,ti OR 'bfr therapy':ab,ti OR 'bfr therapies':ab,ti OR 'therapy, bfr':ab,ti OR 'blood flow restriction exercise':ab,ti OR 'blood flow restriction':ab,ti OR 'blood flow restricted':ab,ti OR 'kaatsu':ab,ti OR 'tourniquets':ab,ti OR 'ischemia':ab,ti OR 'vascular occlusion':ab,ti OR 'occlusion training':ab,ti |
|  | #13 | 'occlusion training':ab,ti |
|  | #12 | 'vascular occlusion':ab,ti |
|  | #11 | 'ischemia':ab,ti |
|  | #10 | 'tourniquets':ab,ti |
|  | #9 | 'kaatsu':ab,ti |
|  | #8 | 'blood flow restricted':ab,ti |
|  | #7 | 'blood flow restriction':ab,ti |
|  | #6 | 'blood flow restriction exercise':ab,ti |
|  | #5 | 'therapy, bfr':ab,ti |
|  | #4 | 'bfr therapies':ab,ti |
|  | #3 | 'bfr therapy':ab,ti |
|  | #2 | 'blood flow restriction therapy':ab,ti |
|  | #1 | 'blood flow restriction training':ab,ti |
| Scopus | #1 | (?TITLE-ABS-KEY?(?"blood Flow Restriction Therapy"? OR? "BFR Therapy"? OR? "BFR Therapies"? OR? "Therapy, BFR"? OR? "blood Flow Restriction Training"? OR? "blood Flow Restriction Exercise"? OR? "blood flow restriction"? OR? "blood flow restricted"? OR? "kaatsu"? OR? "tourniquets"? OR? "ischemia"? OR? "vascular occlusion"? OR? "occlusion training"?)? AND? TITLE-ABS-KEY?(?"overweight"? OR? "obesity"? OR? " adiposity"? OR? "appetite depressants"? OR? "body weight"? OR? "diet, reducing"? OR? "skinfold Thickness"? OR? "lipectomy"? OR? "anti-obesity Agents"? OR? "bariatrics"?)? AND? TITLE-ABS-KEY?(?"randomized controlled trial"? OR? "randomized"? OR? "placebo"? OR? "RCT"?)?)? |
| SPORTDiscus | S1 | TI ( "blood Flow Restriction Therapy" OR "BFR Therapy" OR "BFR Therapies" OR "Therapy, BFR" OR "blood Flow Restriction Training" OR "blood Flow Restriction Exercise" OR "blood flow restriction" OR "blood flow restricted" OR "kaatsu" OR "tourniquets" OR "ischemia" OR "vascular occlusion" OR "occlusion training" ) AND TI ( "overweight" OR "obesity" OR " adiposity" OR "appetite depressants" OR "body weight" OR "diet, reducing" OR "skinfold Thickness" OR "lipectomy" OR "anti-obesity Agents" OR "bariatrics" ) AND TI ( "randomized controlled trial" OR "randomized" OR "placebo" OR "RCT" ) |
| Cochrane Library | #1 | (blood Flow Restriction Therapy):ti,ab,kw OR (BFR Therapy):ti,ab,kw OR (BFR Therapies):ti,ab,kw OR (Therapy, BFR):ti,ab,kw OR (blood Flow Restriction Training):ti,ab,kw (Word variations have been searched) |
|  | #2 | (blood Flow Restriction Exercise):ti,ab,kw OR (blood flow restriction):ti,ab,kw OR (blood flow restricted):ti,ab,kw OR (Kaatsu):ti,ab,kw OR (Tourniquets):ti,ab,kw (Word variations have been searched) |
|  | #3 | (Ischemia):ti,ab,kw OR (vascular occlusion):ti,ab,kw OR (occlusion training):ti,ab,kw (Word variations have been searched) |
|  | #4 | #1 OR #2 OR #3 |
|  | #5 | (Overweight):ti,ab,kw OR (Obesity):ti,ab,kw OR (Adiposity):ti,ab,kw OR (appetite depressants):ti,ab,kw OR (body weight):ti,ab,kw (Word variations have been searched) |
|  | #6 | (diet, reducing):ti,ab,kw OR (skinfold Thickness):ti,ab,kw OR (Lipectomy):ti,ab,kw OR (anti-obesity Agents):ti,ab,kw OR (Bariatrics):ti,ab,kw (Word variations have been searched) |
|  | #7 | #5 OR #6 |
|  | #8 | (randomized controlled trial):ti,ab,kw OR (Randomized):ti,ab,kw OR (Placebo):ti,ab,kw OR (RCT):ti,ab,kw (Word variations have been searched) |
|  | #9 | #4 AND #7 AND #8 |
