# Supplementary material for: Effects of blood flow restriction training on anthropometric and blood lipids in overweight/obese adults: Meta-analysis
Source: Front Physiol. 2022 Nov 29;13:1039591. doi: 10.3389/fphys.2022.1039591 (PMC9745437; doi:10.3389/fphys.2022.1039591)
Supplement: Supplementary file 2 [file Table2.docx]

**Table S2. Basic Information of the Excluded Literatures**

| **No** | **Author** | **Year** | **Title** | **Exclusion reasons** |
| --- | --- | --- | --- | --- |
| 1 | Karabulut M et al | 2015 | Hemodynamic responses and energy expenditure during blood flow restriction exercise in obese population | No target outcome |
| 2 | Golestani, H et al | 2018 | The effect of eight weeks occlusion strength training on oxidative stress responses to a graded maximal exercise test in overweight men | No target outcome |
| 3 | Wang, L et al | 2011 | Effects of different interventions on body mass index and body fat content in overweight and obese adolescents | No target outcome |
| 4 | Barbieri, J. F et al | 2020 | Effect of resistance training with vascular occlusion in the upper limbs | No overweight/obese |
